# Liver cancer: WISP3 suppresses hepatocellular carcinoma progression by negative regulation of β‐catenin/TCF/LEF signalling

**DOI:** 10.1111/cpr.12583

**Published:** 2019-02-22

**Authors:** Hong Gao, Fen‐Fen Yin, Dong‐Xian Guan, Yu‐Xiong Feng, Qian‐Wen Zheng, Xiang Wang, Min Zhu, Xue‐Li Zhang, Shu‐Qun Cheng, Tian‐Wei Chen, Hao Jiang, Er‐Bin Zhang, Jing‐Jing Wang, Qian‐Zhi Ni, Yan‐Mei Yuan, Feng‐Kun Zhang, Ning Ma, Hui‐Jun Cao, Yi‐Kang Wang, Jing‐Jing Li, Dong Xie

**Affiliations:** ^1^ CAS Key Laboratory of Nutrition, Metabolism and Food Safety, Shanghai Institute of Nutrition and Health, Shanghai Institutes for Biological Sciences Chinese Academy of Sciences Shanghai China; ^2^ University of Chinese Academy of Sciences, Chinese Academy of Sciences Shanghai China; ^3^ School of Life Science and Technology ShanghaiTech University Shanghai China; ^4^ Department of Surgery, First People’s Hospital Affiliated Huzhou University Huzhou China; ^5^ Department of General Surgery Fengxian Hospital Affiliated to Southern Medical University Shanghai China; ^6^ Eastern Hepatobiliary Surgery Hospital Second Military Medical University Shanghai China; ^7^ NHC Key Laboratory of Food Safety Risk Assessment China National Center for Food Safety Risk Assessment Beijing China

**Keywords:** hepatocellular carcinoma, metastasis, tumorigenesis, WISP3, β‐catenin

## Abstract

**Objectives:**

Wnt1‐inducible signalling pathway protein 3 (WISP3/CCN6) belongs to the CCN (CYR61/CTGF/NOV) family of proteins, dysregulation of this family contributed to the tumorigenicity of various tumours. In this study, we need to explore its role in hepatocellular carcinoma that remains largely elusive.

**Materials and Methods:**

The expression of WISP3/CCN6 was analysed by qRT‐PCR and Western blotting. Effects of WISP3 on proliferation and metastasis of HCC cells were examined, respectively, by MTT assay and Boyden Chamber. Roles of WISP3 on HCC tumour growth and metastatic ability in vivo were detected in nude mice. Related mechanism study was confirmed by immunofluorescence and Western blotting.

**Results:**

The expression of WISP3 was significantly downregulated in HCC clinical samples and cell lines, and reversely correlated with the tumour size. Forced expression of WISP3 in HCC cells significantly suppressed cell growth and migration in vitro as well as tumour growth and metastatic seeding in vivo. In contrast, downregulation of WISP3 accelerated cell proliferation and migration, and promoted in vivo metastasis. Further study revealed that WISP3 inhibited the translocation of β‐catenin to the nucleus by activating glycogen synthase kinase‐3β (GSK3β). Moreover, constitutively active β‐catenin blocked the suppressive effects of WISP3 on HCC.

**Conclusions:**

Our study showed that WISP3 suppressed the progression of HCC by negative regulation of β‐catenin/TCF/LEF signalling, providing WISP3 as a potential therapeutic candidate for HCC.

## 1 INTRODUCTION

Hepatocellular carcinoma (HCC) is the most common malignant liver cancer, ranking the sixth most common cancer and the second leading cause of cancer‐related death worldwide.^1^ Major risk factors for HCC include chronic hepatitis, alcohol consumption and exposure to other known mutagens.^2^ The incidence and mortality of HCC is higher in China than other countries, due to the infection of hepatitis B virus. Moreover, the prognosis of HCC is very poor, and the 5‐year survival rate worldwide is <5%.^2^

β‐catenin, the central component of the canonical Wnt pathway, plays an important role in cell proliferation, growth, epithelial‐mesenchymal transitions (EMT), cell death and liver regeneration.^3^, ^4^, ^5^ Various molecular and genetic factors, which participated in the aberrant activation of Wnt/β‐catenin pathway, were implicated in tumorigenesis.^4^, ^5^ It has been reported that mutations activating the Wnt/β‐catenin pathway were the most frequent genetic event in human HCC.^6^ Meanwhile, inhibition of β‐catenin signalling exerted anti‐tumour effects on HCC cell lines.^7^, ^8^ Considering that aberrant activation of the β‐catenin signal pathway leads to the tumorigenesis and progression of HCC,^4^ some of the endogenous inhibitors of β‐catenin signal pathway may have potential therapeutic effects.

Wnt1‐inducible signalling pathway protein 3 (WISP3/CCN6) belongs to the CCN (named after CYR61, CTGF and NOV) family of secreted, extracellular matrix (ECM)‐associated signalling matricellular proteins.^9^ Members of CCN family are involved in many vital biological functions, including cell proliferation, adhesion, angiogenesis, ECM production, migration, tumour growth and wound healing,^10^ and dysregulation of this family contributed to the tumorigenicity of various tumours.^11^, ^12^, ^13^ WISP3 has previously been implicated in tumourigenesis and metaplasia in a variety of tissues.^14^, ^15^, ^16^ Moreover, it was reported that β‐catenin dysregulation was partially correlated with inactivating mutations in WISP3 gene in metaplastic carcinomas of the breast.^17^ In microarray‐based screening of differentially expressed genes in HCC, we found that the expression of WISP3 was significantly decreased in HCC samples compared with the adjacent normal tissues (data not shown). In this study, we reported that the expression of WISP3 was significantly decreased in HCC samples and reversely correlated with clinical features of HCC patients, such as AFP level and tumour size. WISP3 suppressed tumour growth, migration and metastasis by negatively regulating β‐catenin/TCF/LEF signalling. Our study has revealed the significance of loss of WISP3 in the progression of HCC and therefore provides a potential drug candidate in HCC therapy.

## 2 MATERIALS AND METHODS

### 2.1 HCC tissue samples and cell lines

Seventy‐one pairs of primary HCC and their adjacent normal tissues, which were at least 3‐4 cm away from cancer, were obtained from HCC patients treated at Eastern Hepatobiliary Surgery Hospital during 2008‐2012 with written and informed consent. The fresh specimens were stored at −80°C until analysis. Our work was approved by the Institutional Review Board of the Institute for Nutritional Sciences, Chinese Academy of Sciences. Normal liver cell lines QSG‐7701 and LO2, as well as liver cancer cell lines Huh7 and SK‐HEP‐1, were purchased from Cell Bank of Type Culture Collection of Chinese Academy of Sciences, Shanghai Institute of Cell Biology, Chinese Academy of Sciences. Hep3B cells were gift from Dr Hui Wang (Shanghai Institutes for Biological Sciences, Chines Academy of Sciences). The portal vein tumour thrombus 1 (PVTT‐1) cell line was established in our laboratory.^18^, ^19^

### 2.2 Reagents and antibodies

Antibodies to WISP3, cyclin D1, α‐tubulin, β‐catenin, CTGF and GAPDH were obtained from Santa Cruz Biotechnology Inc Antibody to MMP7 was obtained from Bioworld Technology (Nanjing, China). Antibodies to p62, Glycogen synthase kinase 3β (GSK3β), phosphorylated GSK3β (pSer9), SAPK/JNK and Phospho‐SAPK/JNK (Thr183/Tyr185) were purchased from BD Transduction Laboratories (San Diego, CA, USA). Antibodies to E‐cadherin, Akt, phosphorylated Akt (pSer473, pThr308), Snail and horseradish peroxidase‐conjugated secondary antibodies were purchased from Cell Signaling Technology (Danvers, MA, USA). Trizol reagent was purchased from Invitrogen (Waltham, MA, USA). Luciferin was purchased from Xenogen Biotechnology (Shanghai, China).

### 2.3 RNA extraction and real‐time polymerase chain reaction analysis

Total RNA was isolated from cell lines and tissues using Trizol reagent (Invitrogen). A total of 2 µg RNA with high quality was processed directly to cDNA with the reverse transcription kit (Promega, Madison, WI, USA), following the manufacturer's instructions, in a total volume of 25 µL. The primer sequences were (5′‐3′): WISP3‐F: GGAGCGTGGGGTTTGCAGAGG and WISP3‐R: GCCCTGTACCCTGCAGCAGAACTGT; β ‐actin‐F: GATCATTGCTCCTCCTGAGC and β‐actin‐R: ACTCCTGCTTGCTGATCCAC. Amplification reactions were performed in a 15 µL volume of the LightCycler‐DNA Master SYBR Green I mix (Roche Applied Science, Penzberg, Germany) with 10 pmol/L of primer, 2 mmol/L MgCl_2_, 200 µmol/L deoxynucleotide triphosphate mixture, 0.5 units of Taq DNA polymerase and universal buffer. All of the reactions were performed in triplicates in Mx3000 system (Stratagene), and the thermal cycling conditions were as follows: 95°C for 3 minutes; 40 cycles of 95°C for 30 seconds, 60°C for 20 seconds and 72°C for 20 seconds; 72°C for 10 minutes. The relative mRNA level of WISP3 was normalized to that of β‐actin.

### 2.4 Construction of plasmids and transfection

The coding sequence for the full length of WISP3 was cloned into the pcDNA3.1‐Myc plasmid (Invitrogen) to generate the WISP3 expression vector. Then, the WISP3 coding sequence and the myc tag were subcloned into pRDI‐292 lentiviral expression vector, and cells were selected with puromycin. The coding sequence for β‐catenin with substitute mutations of T41A and S45A was cloned into pcDNA3.1‐Myc. The resulting plasmid was transfected into cells by FuGENE6 (Roche, Basel, Switzerland), and the transfected cells were selected in the presence of G418. Resistant clones were further confirmed the expression of β‐catenin by Western blot. The coding sequence for luciferase was cloned into FG12 expressing vector. PVTT‐1 and QSG‐7701 cells were labelled with luciferase for metastasis assay.

### 2.5 Construction of shRNA targeted WISP3

ShRNA specially targeting WISP3 was constructed as follows: the complete coding sequence of human WISP3 was retrieved from the NCBI GenBank database, and two paired insert DNA fragments according to the coding regions of WISP3, starting at 232 and 331, were designed using software offered by Qiagen (Valencia, CA, USA). The following short anti‐wisp3 RNA oligonucleotides were synthesized: shWISP3 6# 5′‐gatccACCTCAGCGTAAACAGTTtttcaagagaAAACTGTTTACGCTGAGGTTTTTTTCTCGAGg‐3′ and shWISP3 8# 5′‐ gatccGCTGTAAAATCTGTGCCAAGttcaagagaCTTGGCACAGATTTTACAGTTTTTTCTCGGg‐3′. A control sequence, which was not specific for WISP3 (shWISP3 con), was also constructed: 5′‐gatccGGCGCATAAGAAGCATATAttcaagagaTATATGCTTCTTATGCGCCTTTTTTg‐3′. Sequences were evaluated for specificity by a BLAST search. Annealed oligonucleotides were ligated with the Linear SIREN‐RetroQ vector, resulting in the shWISP3 6#, shWISP3 8# and shWISP3 control recombinants. The recombinants were identified by restriction endonuclease analysis and DNA sequencing. The vector contains a puromycin resistance gene for the selection of stable transfection. Transfected cells were selected with puromycin, and the expression of WISP3 in the resistant clones was further confirmed by Western blot.

### 2.6 MTT assay

The in vitro cell growth was measured by MTT assay. Cells were plated in 96‐well plates with a concentration of 1000 cells per well. To measure cell growth, 20 µL 5 mg/mL MTT (3‐(4, 5‐Dimethylthiazol‐2‐yl)‐2, 5‐diphenyl‐tetrazolium bromide) was added into the medium and cultured at 37°C for 4 hours. Then, the medium was removed, 200 µL DMSO was added to dissolve the generated formazan, and the OD550 value of the solvent was measured by an automatic microplate reader. The measurement process was performed every 24 hours for 7 days to generate a cell growth curve.

For experiments with recombinant human WISP3 (rhWISP3), Huh7 and PVTT‐1 cells were plated in 96‐well plates with a concentration of 1500 cells per well for 24 hours followed by serum starvation for 16 hours. Cells were treated with 400 ng/mL of rhWISP3 (Peprotech, Rocky Hill, NJ, USA) or with diluent in 10% FBS‐DMEM medium for 6 days. The cell growth curve was generated using the MTT assay.

### 2.7 In vivo tumorigenicity assay

All procedures were performed in agreement with SIBS Guide for the Care and Use of Laboratory Animals and approved by Animal Care and Use Committee, Shanghai Institutes for Biological Sciences. Male nude mice were housed under standard conditions. Cell suspensions (2 × 10^5^) of Huh7/vec or Huh7/WISP3 cells in a total volume of 100 µL were injected subcutaneously into the 4‐week‐old nude mice (four mice for each group, randomly assigned). On the 28th day after inoculation, the tumours were harvested, photographed and weighted.

### 2.8 In vivo metastasis assay

Cells (5 × 10^5^ for PVTT‐1 and 5 × 10^6^ for QSG‐7701) labelled with luciferase were injected into the left ventricle of 6‐week‐old nude mice (five mice for each group, randomly assigned). The metastatic lesions were monitored weekly by luciferase expression, which was determined by an in vivo Living Image System (Xenogen, Alameda, CA, USA). Before mice were anesthetized with isoflurane (Abbott, Abbott Park, IL, USA), an aqueous solution of luciferin (Xenogen, 150 mg/kg intraperitoneally) was injected 10 minutes prior to imaging. The mice were placed into a light‐tight chamber of the CCD camera system (Xenogen), and the photons emitted from the luciferase‐expressing cells within the animal were quantified for 1 minutes using the software program Living Image as an overlay on Igor (Wavemetrics, Seattle, WA, USA).

### 2.9 Cell migration assay

The migration assay was carried out using a 10‐well Boyden Chamber (Neuro Probe, Gaithersburg, MD, USA) with a polycarbonate membrane of 8 µm pore size. Briefly, the underside of the filter was coated with 50 µg/mL rat tail type I collagen at 4°C overnight. Cells (2 × 10^5^) in 1% foetal bovine serum were plated in the upper wells. Medium with 10% FBS was used as a chemoattractant in the bottom wells. The chamber was then incubated at 37°C for 6‐8 hours. Cells that did not migrate through the pores of the filter were removed. Cells that migrated to the underside of the filter were stained with eosin and photographed using an inverted microscope. Six fields of each well were counted, and the experiments were repeated at least three times.

For experiments with recombinant human WISP3 (rhWISP3), Huh7 and PVTT‐1 cells (2 × 10^5^) with 400 ng/mL of rhWISP3 (Peprotech, Rocky Hill, NJ, USA) or with diluent in 1% foetal bovine serum were plated in the upper wells. The other steps were the same with above description of cell migration assay.

### 2.10 Western blotting

Cells or HCC samples were lysed in RIPA buffer for 30 minutes on ice, and the lysates were centrifuged at 10 000 *g* for 15 minutes at 4°C. Protein concentration was determined using the Bradford reagent (Sigma, St Louis, MO, USA) according to the manufacturer's instructions. Equal amounts of total cellular protein were mixed with loading buffer and subjected to 10% sodium dodecyl sulphate/polyacrylamide gel electrophoresis. Proteins were transferred to polyvinylidene difluoride membranes (Millipore, Bedford, MA, USA) and immunoblotted with specific antibodies. The immunoreactive protein bands were visualized using an enhanced chemiluminescence kit (Pierce, Rockford, IL, USA). ImageJ software was used for quantification of Western blots.

### 2.11 Immunofluorescence

Cells were digested with trypsin and plated on the slides. Twenty‐four hours later, cells were washed thrice with phosphate‐buffered saline (PBS) at room temperature, fixed in methanol at −20°C for 5 minutes, blocked with 1% bovine serum albumin at room temperature for 1 hour and then incubated with the primary antibody overnight at 4°C. After washing three times in PBS, cells were incubated with the secondary antibody (1:1000) at room temperature for 1 hour. Cells were washed three times with PBS for 5 minutes each in darkness, and the nuclei were stained with Hoechst. Fluorescence was monitored by an inverted confocal laser microscopy (Carl Zeiss, New York, NY, USA).

### 2.12 Nuclear protein extraction

The cytoplasmic and nuclear proteins were prepared as described previously.^20^ Briefly, cells were washed with ice‐cold PBS and lysed in buffer containing 1% Nonidet P‐40, 10 mmol/L HEPES/potassium hydroxide, pH 7.9, 1 mmol/L dithiothreitol, 0.1 mmol/L ethylene glycol‐bis (β‐aminoethylether)‐N,N,N^,^,N^,^‐tetraacetic acid, 0.1 mmol/L EDTA and 0.5 mmol/L phenylmethylsulfonyl fluoride, followed by centrifugation at 1000 *g* for 5 minutes to pellet the nuclei. After separation of the cytoplasmic fraction, nuclei were resuspended in ice‐cold buffer containing 20 mmol/L HEPES/potassium hydroxide, pH 7.9, 0.4 mmol/L sodium chloride, 1 mmol/L dithiothreitol and 0.2 mmol/L phenylmethylsulfonyl fluoride; incubated for 1 hour on ice; and then centrifuged to clear the cellular debris. The expression of β‐catenin in nucleus and cytoplasm was determined by Western blot.

### 2.13 Luciferase reporter assay

Cells were plated at a subconfluent density and cotransfected with 0.05 µg of the reporter plasmid, 0.5 µg of expression vectors and 0.02 µg of Renilla luciferase pRL‐TK (internal control for transfection efficiency). Cell lysates were prepared 24 hours after transfection, and the reporter activity was measured using the Dual‐Luciferase Reporter Assay System (Promega, Madison, WI, USA). Transfections were performed in triplicate and repeated three times to ensure reproducibility.

### 2.14 Preparation of conditioned medium and cell treatment

Conditioned medium (CM) from confluent cultures of QSG‐7701 cells stably transfected with either the control (WISP3 abundant) or shRNA targeted WISP3 vector (WISP3 scarcity) was collected, centrifuged at 600 *g* for 10 minutes, filtered, sterilized and stored at −70°C for use. Huh7 cells were treated with the CM 1:1 diluted with the fresh medium. After incubation for 24 hours, cell migration assay was performed as described above.

### 2.15 Statistical analysis

All data are presented as mean ± SE. Statistical analysis was performed using SPSS 13.0 (SPSS Inc., Chicago, IL, USA). Statistically significant differences were determined by Student's *t *test, and one‐way ANOVA test. Differences between groups were considered statistically significant at *P* < 0.05.

## 3 RESULTS

### 3.1 WISP3 is downregulated in HCC cell lines and clinical samples, and correlates with the clinical features

**Figure 1:**
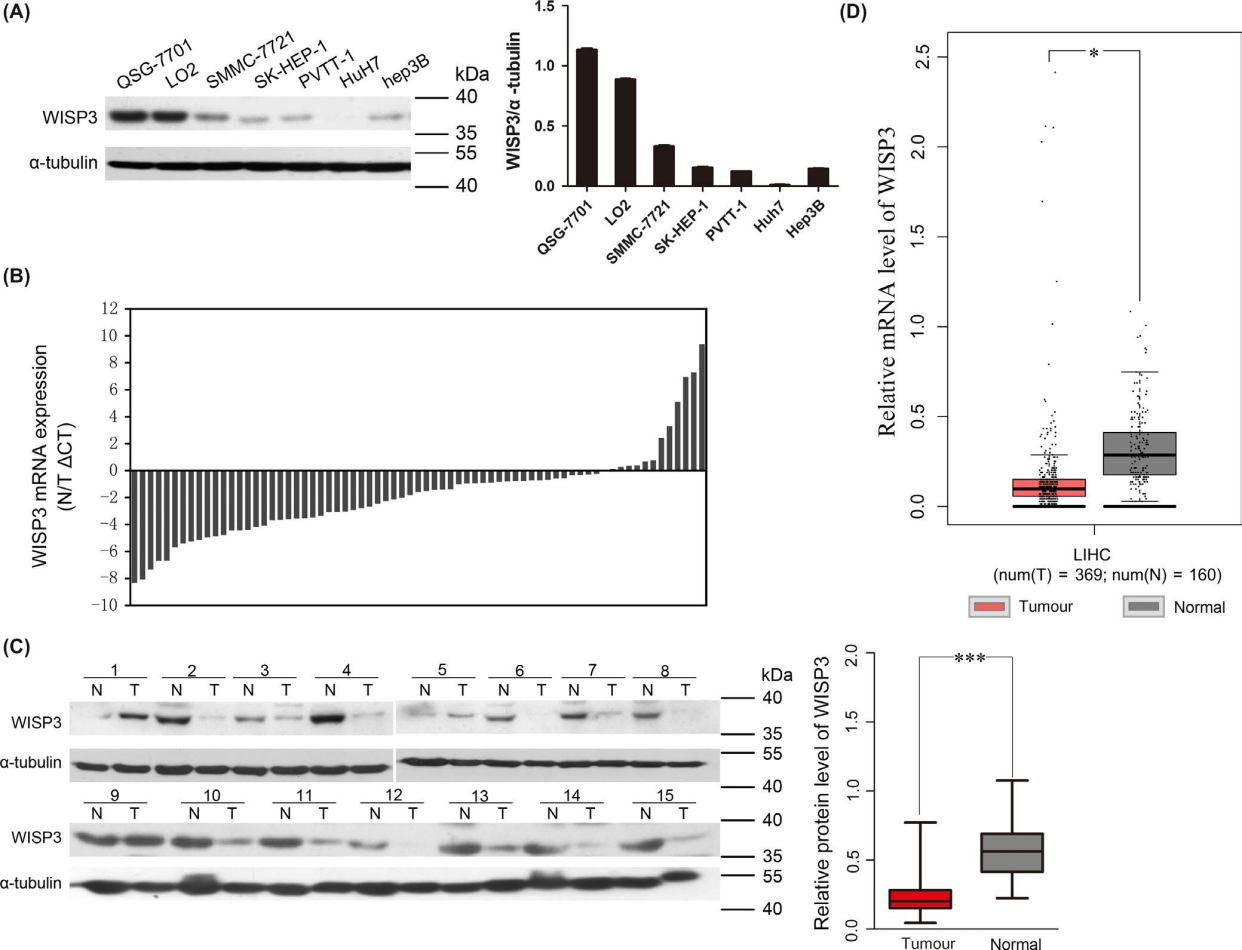
The expression of WISP3 was decreased in HCC cell lines and clinical samples. A, The protein level of WISP3 in two normal liver cell lines (QSG‐7701 and LO2) and five liver cancer cell lines (SMMC‐7721, SK‐HEP‐1, PVTT‐1, Huh7 and Hep3B) was examined by Western blot. Left panel: Result of Western blot. Right panel: Grey analysis results of the Western blot bands. B, The mRNA level of WISP3 in 71 HCC samples and paired normal liver tissues was detected by quantitative real‐time PCR. The expression of WISP3 was normalized to β‐actin. T, tumour samples; N, matched normal tissues. C, Protein level of WISP3 in 15 randomly chosen HCC samples and paired normal tissues; α‐tubulin was used as loading control. Left panel: Result of Western blot. Right panel: Grey analysis results of the Western blot bands. D, The mRNA levels of WISP3 in liver cancer samples in comparison with normal liver samples derived from GEPIA. *P < 0.05， ***P < 0.001 vs normal liver tissue

To explore the potential roles of WISP3 in HCC, we first examined its expression in normal hepatic cell lines (QSG‐7701 and LO2) and HCC cell lines (SMMC‐7721, SK‐HEP‐1, PVTT‐1, Huh7, Hep3B) by Western blot (Figure 1A). The expression level of WISP3 was significantly decreased in the invasive cell lines (PVTT‐1 and Huh7) compared with the normal hepatic cells (QSG‐7701 and LO2). Then, we examined mRNA level of WISP3 in 71 clinical HCC samples and their matched normal liver tissues by real‐time PCR. Downregulation of WISP3 mRNA was observed in 83% (59/71) HCC tissues compared with their adjacent normal tissues (Figure 1B). Results of Western blotting further confirmed that the protein level of WISP3 also significantly decreased in HCC samples (Figure 1C). In addition, downregulation of WISP3 in liver cancer samples compared with normal liver samples was further confirmed by an online Bioinformatics tool (GEPIA, Figure 1D). The correlation between WISP3 mRNA levels and pathological characteristics was analysed, and we found that the decreased level of WISP3 mRNA was correlated with larger tumour size and higher AFP level (Table S1). These results suggested that WISP3 was dysregulated in clinical HCC patients and indicated its suppressive role in the progression of HCC.

### 3.2 Overexpression of WISP3 in HCC cells suppresses cell growth and migration in vitro as well as tumorigenesis and metastasis in vivo

**Figure 2:**
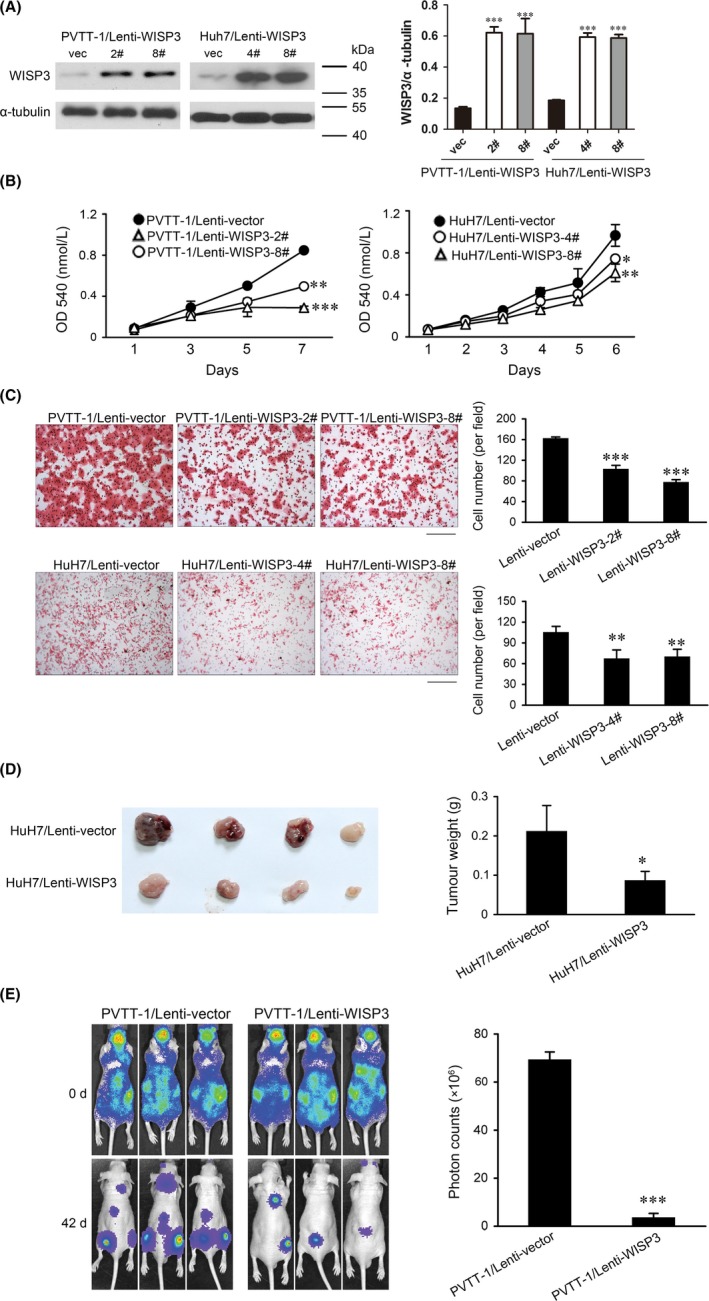
Forced expression of WISP3 inhibited cell growth, migration and tumorigenesis. A, Overexpression of WISP3 in PVTT‐1 and Huh7 cells. Cells were infected with lentivirus expressing WISP3 or vector control, followed by selection with puromycin. Lysate was examined by Western blot. α‐tubulin was used as a loading control. B, The effects of WISP3 on the proliferation of PVTT‐1 and Huh7 cells were measured by MTT assay. C, The effects of WISP3 on the migration of PVTT‐1 and Huh7 cells were measured by Boyden Chamber assay. *Left photograph*: Boyden Chamber assay of PVTT‐1 and Huh7 cell lines after forced expression of WISP3. *Right photograph*: calculation of cells that migrated through the filter following eosin staining. Scale bar = 200 μm. D, The effects of WISP3 on growth of Huh7 in vivo. *Left photograph*: representative photograph of the xenografts. *Right photograph*: tumour weight. E, Overexpression of WISP3 inhibited HCC cell growth in distant organs. WISP3 overexpressing cells and control cells labelled with luciferase were injected into the left ventricle of nude mice. The metastatic lesions were measured by bioluminescent signals on days 0 and 28 (five mice for each group). Results were presented as mean ± SE of three independent experiments. *P < 0.05, **P < 0.01, ***P < 0.001 vs Lenti‐vector cells

To investigate the function of WISP3 in HCC, WISP3 was stably overexpressed in PVTT‐1 and Huh7 cells, and the expression was confirmed by Western blot (Figure 2A). Since the expression of WISP3 was reversely correlated with tumour size, we first examined the effect of WISP3 on cell growth by MTT assay. As shown in Figure 2B, overexpression of WISP3 remarkably decreased cell growth in both PVTT‐1 and Huh7 cells. In addition, forced expression of WISP3 significantly suppressed cell migration examined by Boyden Chamber assay, an important process involved in tumour metastasis (Figure 2C). Since WISP3 is an ECM proteins, we examined the effects of recombinant human WISP3 on HCC cell growth and migration. As shown in Figure S1A,B, rhWISP3 (400 ng/mL) significantly inhibited the growth and migration of Huh7 and PVTT‐1.

Consistent with in vitro study, forced expression of WISP3 in Huh7 cells (Huh7/WISP3) dramatically retarded tumour growth in nude mice compared with the control cells (Huh7/vec) in xenograft mouse model (Figure 2D). To further examine the effects of WISP3 on HCC metastasis in vivo, cells were labelled with luciferase and injected into the left ventricles of nude mice. The metastatic seeding was monitored by bioluminescent signals. Compared with the control cells, cells overexpressing WISP3 developed fewer and smaller metastatic lesions (Figure 2E), which was consistent with the in vitro observations. These results indicated that overexpression of WISP3 in HCC cells inhibited cell growth and migration in vitro as well as tumorigenicity and metastasis in vivo.

### 3.3 Knockdown of WISP3 in HCC cells promotes cell growth and migration in vitro as well as tumorigenesis and metastasis in vivo

**Figure 3:**
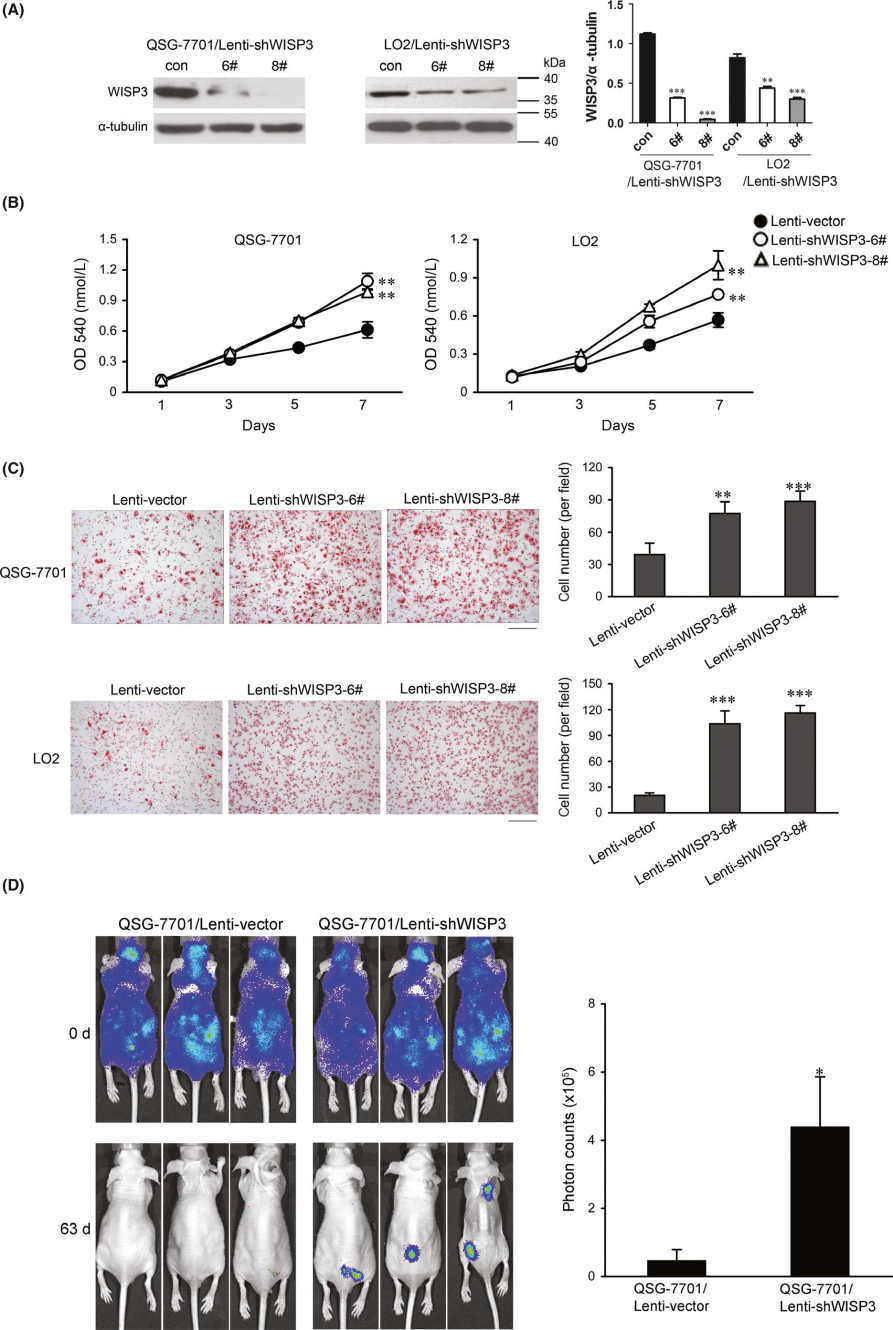
Knockdown of WISP3 promoted cell growth and migration, as well as metastasis in vivo. A, Silencing the expression of WISP3 in QSG‐7701 and LO2 cells. Left panel: Result of Western blot. Right panel: Grey analysis results of the Western blot bands. B, Knockdown the expression of WISP3 promoted the growth of QSG‐7701 and LO2 cells by MTT assay. C, Knockdown the expression of WISP3 promoted the migration of QSG‐7701 and LO2 cells by Boyden Chamber assay. *Left photograph*: Boyden Chamber assay of QSG‐7701 and LO2 cell lines after knockdown the expression of WISP3. *Right photograph*: calculation of cells that migrated through the filter following eosin staining. Scale bar = 200 μm. D, Knockdown of WISP3 promoted cell growth in distant organs. The metastatic lesions were measured by bioluminescent signals on days 0 and 63 (five mice for each group). Results were presented as mean ± SE of three independent experiments. *P < 0.05, **P < 0.01, ***P < 0.001 vs Lenti‐vector cells

To further elucidate the role of WISP3 in HCC cell growth and migration, we employed siRNA (shWISP3 6# and shWISP3 8#) to knock down the expression of endogenous WISP3 in QSG‐7701 and LO2 cells (Figure 3A). Silencing the expression of WISP3 in QSG‐7701 and LO2 cells dramatically stimulated cell proliferation and migration (Figure 3B,C). Furthermore, knockdown of WISP3 facilitated metastasis of QSG‐7701 cells to distant organs (Figure 3D). Taken together, these results suggested that WISP3 acted as a negative regulator in the progression of HCC.

### 3.4 WISP3 inhibits cell growth and migration by negatively regulating Akt/GSK3β/β‐catenin/TCF signalling

To identify the underlying molecular mechanism for the suppressive effects of WISP3 in HCC, we firstly screened several signalling pathways with luciferase reporter assays. We found that WISP3 remarkably inhibited the activity of TOPflash, a classic Wnt‐responsive reporter in HEK293T, as well as HCC cell Huh7 and PVTT‐1. As shown in Figure 3A and Figure S2A, WISP3 repressed the transcriptional activity of β‐catenin/TCF in a dose‐dependent manner, while WISP3 had very little effect on the activation of ATF2 luciferase reporter in both HEK293T and HCC cells (Figure S2B‐D). No significant differences in the mRNA level of β‐catenin were detected in WISP3 overexpressing and knockdown cells compared to control (Figure S2E,F). It was known that β‐catenin was regulated at multiple levels including stabilization/translocation.^21^ Then, we examined the localization of β‐catenin. As shown in Figure 3B, β‐catenin mainly localized in the cytoplasm and nucleus in the highly invasive PVTT‐1 and Huh7 cells (Figure 3B), whereas the membrane localization of β‐catenin was dominant in the normal hepatic cells QSG‐7701 and LO2 (Figure 3C). However, overexpression of WISP3 in PVTT‐1 and Huh7 resulted in an increase of β‐catenin in membrane and a decrease of β‐catenin in the nucleus. In contrast, downregulation of WISP3 resulted in the decrease of membrane‐localized β‐catenin and the increase of intracellular β‐catenin in the cytoplasm and nucleus (Figure 3C). These observations were further confirmed by the nuclear extraction. In addition, WISP3 influenced the expression of canonical Wnt/β‐catenin downstream genes, including cyclin D1,^22^ matrix metalloproteinases‐7(MMP7),^23^ Snail^24^ and CTGF,^25^ while had no effect on the phosphorylation of JNK^26^, ^27^ (Thr183/Tyr185), which was a non‐canonical Wnt/PCP pathway downstream molecule (Figure 3B‐D). These data suggested that WISP3 affected the canonical Wnt//β‐catenin pathway rather than non‐canonical Wnt/PCP pathway. Furthermore, as shown in Figure S5, cells treated with WISP3 CM led to inhibition of cell migration and suppression of β‐catenin/TCF transcriptional activity through downregulating the protein level of nuclear β‐catenin. Taken together, these results suggested that WISP3 negatively regulated β‐catenin/TCF signalling.

Glycogen synthase kinase 3β is a key negative regulator of β‐catenin.^3^, ^4^, ^5^ PI3K/Akt was reported to act upstream of GSK3β. Therefore, we examined whether WISP3 affected the activation of Akt and GSK3β. As shown in Figure 3D, forced expression of WISP3 dramatically abrogated phosphorylation of Akt (Thr308 and Ser473) and GSK3β. As expected, downregulation of WISP3 enhanced the phosphorylation of Akt and increased the inhibition‐associated phosphorylation of GSK3β on Ser‐9. Taken together, the phosphorylation of Akt/GSK3β may involve in inhibition of nuclear accumulation of β‐catenin by WISP3.

**Figure 4:**
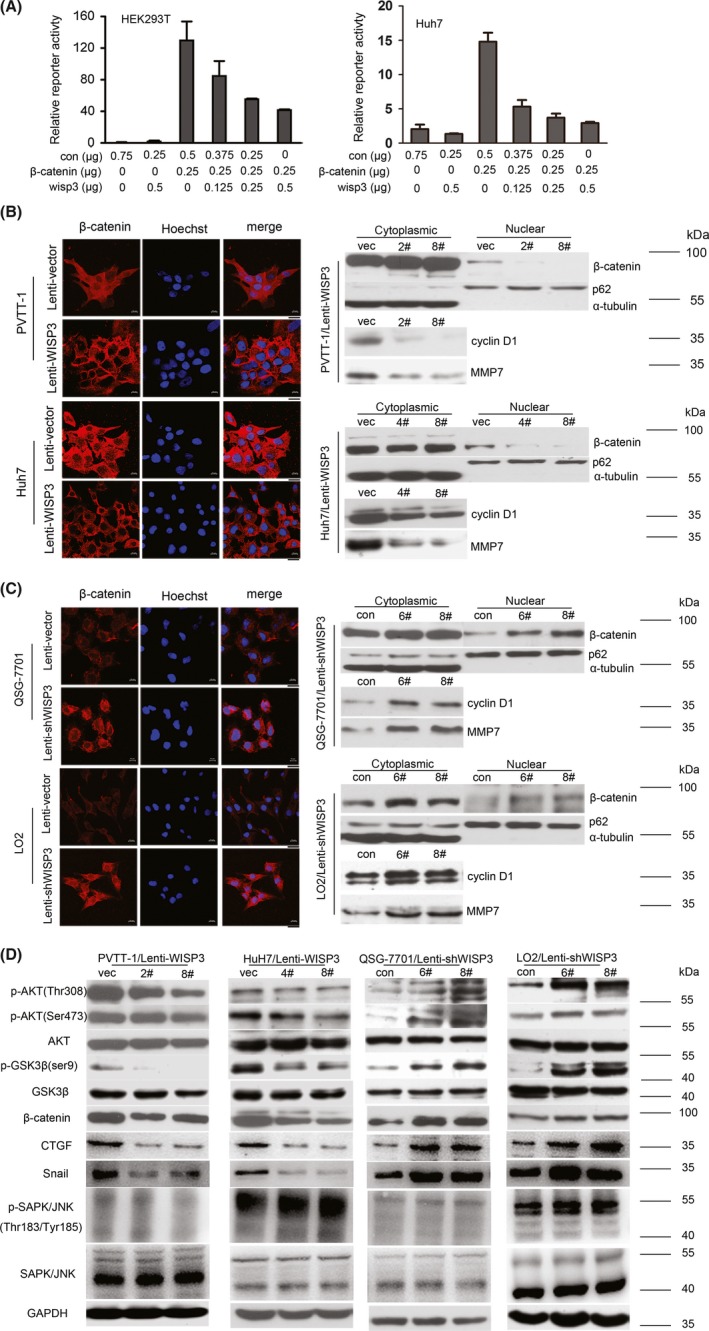
WISP3 repressed β‐catenin/TCF transcriptional activity by inhibition of β‐catenin nuclear accumulation. A, WISP3 inhibited the activity of TOPflash reporter in a dose‐dependent manner in HEK293T cells. Left panel: WISP3 inhibited the activity of TOPflash reporter in a dose‐dependent manner in HEK293T cells. Right panel: WISP3 inhibited the activity of TOPflash reporter in a dose‐dependent manner in Huh7 cells. B, Overexpression of WISP3 inhibited the nuclear localization of β‐catenin. *Left photograph*: representative photomicrographs of PVTT‐1/Lenti‐WISP3 stable cells, and Huh7/Lenti‐WISP3 stable cells immunostained with anti‐β‐catenin antibody (red). Cell nuclei were counterstained with Hoechst (blue). *Right photograph*: protein level of both cytoplasmic and nuclear β‐catenin was further confirmed by Western blot. The targeted genes of β‐catenin/TCF signalling, cyclin D1 and MMP7 were detected. Scale bar = 20 μm. C, Knockdown of WISP3 promoted β‐catenin nuclear localization. *Left photograph*: representative photomicrographs of QSG‐7701/Lenti‐shWISP3 stable cells, and LO2/Lenti‐shWISP3 stable cells. *Right photograph*: translocation of β‐catenin and its target genes were further confirmed by Western blot. Scale bar = 20 μm. D, WISP3 regulated the phosphorylation of Akt/GSK3β. The effects of WISP3 on the phosphorylation of Akt and GSK3β were examined in PVTT‐1, Huh7, QSG‐7701, LO2 cells.

We next examined whether the effects of WISP3 on the progression of HCC were β‐catenin/TCF‐dependent. Constitutively active β‐catenin (CA β‐catenin) was introduced into Huh7 cells (Figure 4A). As shown in Figure 4B,C, WISP3‐induced inhibitory effects on cell growth and migration were rescued by CA β‐catenin. These observations indicated that the suppressive effects of WISP3 were β‐catenin/TCF signalling‐dependent.

## 4 DISCUSSION

CCN proteins influence multiple physiological and pathological processes, mounting evidence indicated that abnormal levels of CCN proteins played different roles in tumorigenesis, either enhance or inhibit cancer progression.^13^, ^28^, ^29^ WISP3 was frequently studied in breast cancer. It was reported that WISP3 was downregulated in 80% of the aggressive inflammatory breast cancers with high metastatic potential.^15^ Low CCN6 protein levels were associated with upregulated expression of phospho‐Akt‐1 (Ser473) expression in 21% of invasive breast carcinomas.^30^ Mammary epithelial cell‐specific *ccn6* (WISP3) knockout mice develop invasive high‐grade mammary carcinomas,^31^ suggesting CCN6 as a tumour suppressor in breast cancer. Nonetheless, WISP3 showed distinct effects in colorectal carcinomas,^32^ gastric cancer^33^ and bladder cancer.^34^ Report about the roles of WISP3 in HCC is still lacking.

In our study, we demonstrated that WISP3 was significantly downregulated in HCC tissues compared with their matched normal counterparts. The expression levels of WISP3 were correlated with the pathologic characteristics of the patients, such as tumour size and AFP level. Analysis of WISP3 expression in HCC cell lines demonstrated that the expression of WISP3 reversely correlated with the invasive capabilities of HCC cells. The expression pattern of WISP3 in both cell lines and clinical samples suggested that it might play a suppressive role during the progression of HCC. By manipulating the expression level of WISP3 in HCC cell lines, we demonstrated that WISP3 regulated cell growth as well as migration. Moreover, results from in vivo tumorigenicity assay and metastasis seeding assay further confirmed the significance of WISP3 in the development of HCC. Taken together, our investigation implied that WISP3 acted as a negative regulator of HCC progression.

Among the mechanisms leading to the malignant transformation of liver cells, hyperactivation of β‐catenin/TCF signalling was very common.^35^ WISP genes were first described as downstream targets of the Wnt signalling pathway,^9^ whereas the β‐catenin/TCF/LEF signalling activity was regulated by the WISP family. It has been reported that WISP1 auto‐regulated its expression through regulation of β‐catenin phosphorylation and nuclear translocation.^11^ Also, our results suggested WISP3 could affect the subcellular localization of β‐catenin. In the unstimulated cells, Serine‐45 in the β‐catenin protein was constitutively phosphorylated by CK1 and GSK3β, preventing β‐catenin accumulation in the cytoplasm and the nucleus. During the inhibition of GSK3β induced by Wnt activation, β‐catenin was accumulated and translocated to the nucleus to regulate the transcription of Wnt target genes.^3^, ^4^, ^5^ We demonstrated that the overexpression of WISP3 was associated with decreased phosphorylation of Akt and GSK3β as well as the reduced expression of β‐catenin in the nucleus. In contrast, knockdown of endogeneous WISP3 enhanced phosphorylation of Akt, leading to increased phosphorylation of GSK3β and free cytoplasmic pool of β‐catenin, which facilitated its nuclear translocalization and transcriptional function. The nuclear β‐catenin induced a gene expression pattern favouring tumour metastasis, including cyclin D1 and MMP7,^23^ which can promote cell proliferation and invasion. Furthermore, the suppressive effects of WISP3 on HCC were rescued by the constitutively active β‐catenin, proving that the function of WISP3 was mediated by β‐catenin/TCF signalling. Taken together, our results suggested WISP3, a downstream target of the Wnt signalling pathway, might negatively regulate β‐catenin signalling through an endogenous feedback. However, loss of WISP3 during HCC progression may lead to shutdown of this negative feedback, thus result in constitutive activation of β‐catenin.

E‐cadherin andβ‐catenin are important epithelial adhesion molecules in normal epithelium,^36^ and β‐catenin plays a crucial role in cell‐cell adhesion mediated by E‐cadherin.^21^ A disturbance in epithelial cell adhesion, which leads to a more invasive and metastatic phenotype, is a hallmark of tumour progression.^21^ Our result also indicated that overexpression of WISP3 induced mesenchymal‐epithelial transition (Figure S6A), and enhanced the expression of E‐cadherin in Huh7 and PVTT‐1 (Figure S6B). Consistently, it has been reported that WISP3 knockdown activates growth factor‐independent survival that requires activation of the PI3K/Akt signalling pathway^37^ and regulation of E‐cadherin.^38^ Loss of E‐cadherin liberated β‐catenin from the membrane, which resulted in accumulation of β‐catenin in the nuclear and made cells more motile and invasive.^39^ Our results suggested that WISP3 might alternatively influence the subcellular localization of β‐catenin by regulating the expression of E‐cadherin which recruited β‐catenin to adherens junctions and then inhibited β‐catenin/TCF signalling. Taken together, reduction of WISP3 in HCC contributed to downregulation of E‐cadherin and inhibition of GSK3β, both of which lead to the accumulation and activation of β‐catenin in nucleus.

In summary, our study has shown that the expression of WISP3 was significantly decreased in clinical samples of HCC. Silencing the expression of WISP3 accelerated cell growth and migration, whereas forced expression of WISP3 suppressed tumour growth and metastasis seeding. All of these findings provide the first evidence that WISP3 exerts a strong inhibitory effect in human HCC. The aberrant activation of β‐catenin signal pathway occurs frequently in HCC and many other types of solid tumour. Wnt inhibitors in the extracellular compartment prevent the aberrant β‐catenin signalling by regulating the accessibility of the Wnt ligands to Frizzled receptors.^35^

In this context, WISP3, an ECM protein, might be a novel therapy candidate to retard the development and metastasis of HCC by inhibiting β‐catenin signalling.

## CONFLICT OF INTEREST

The authors declare that they have no conflict of interest.

## AUTHORS’ CONTRIBUTIONS

Hong Gao and Fen‐Fen Yin contributed to the conception and design, collection and assembly of data, and wrote the manuscript; all authors were involved in final approval of the submitted and published versions.

5

**Figure 5:**
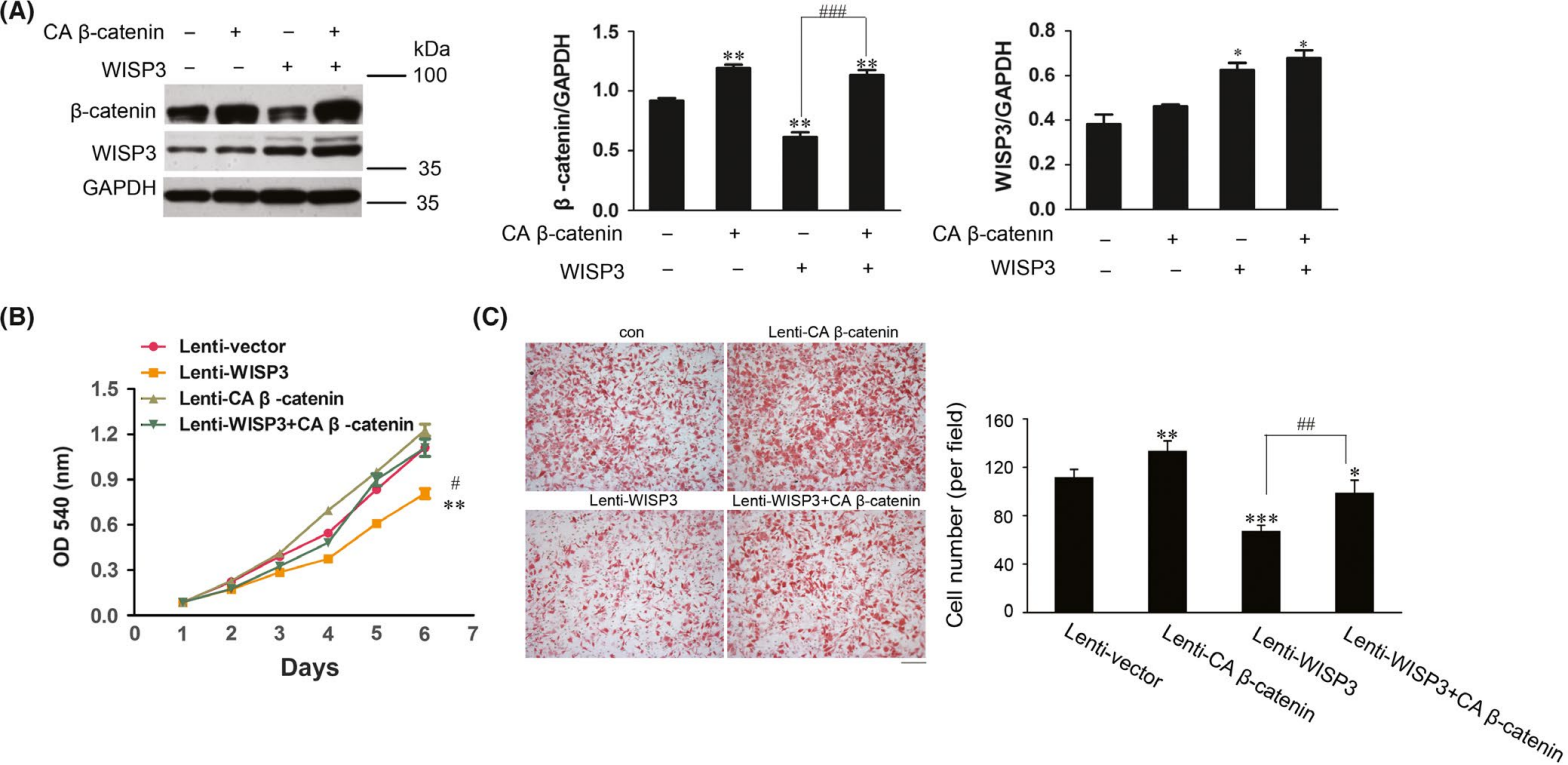
Constitutively active β‐catenin (CA β‐catenin) rescued the suppressive effects of WISP3. A, The expression levels of β‐catenin and WISP3 were examined by Western blot. Left panel: Result of Western blot. Medium and Right panel: Grey analysis results of the Western blot bands. B, CA β‐catenin rescued the growth suppression induced by WISP3. C, CA β‐catenin rescued the migration inhibition effect induced by WISP3. *Left photograph*: Boyden Chamber assay of Huh7. *Right photograph*: calculation of cells that migrated through the filter following eosin staining. Scale bar = 200 μm. Results were presented as mean ± SE of three independent experiments. *P < 0.05, **P < 0.01, ***P < 0.001 vs Lenti‐vector cells; ##*P* < 0.01, ###*P* < 0.001 vs Lenti‐WISP3+CA β‐catenin cells. Scale bar = 200 μm
