# Supplementary figures and images for: Liver cancer: WISP3 suppresses hepatocellular carcinoma progression by negative regulation of β‐catenin/TCF/LEF signalling
Source: Cell Prolif. 2019 Feb 22;52(3):e12583. doi: 10.1111/cpr.12583 (PMC6536422; doi:10.1111/cpr.12583)

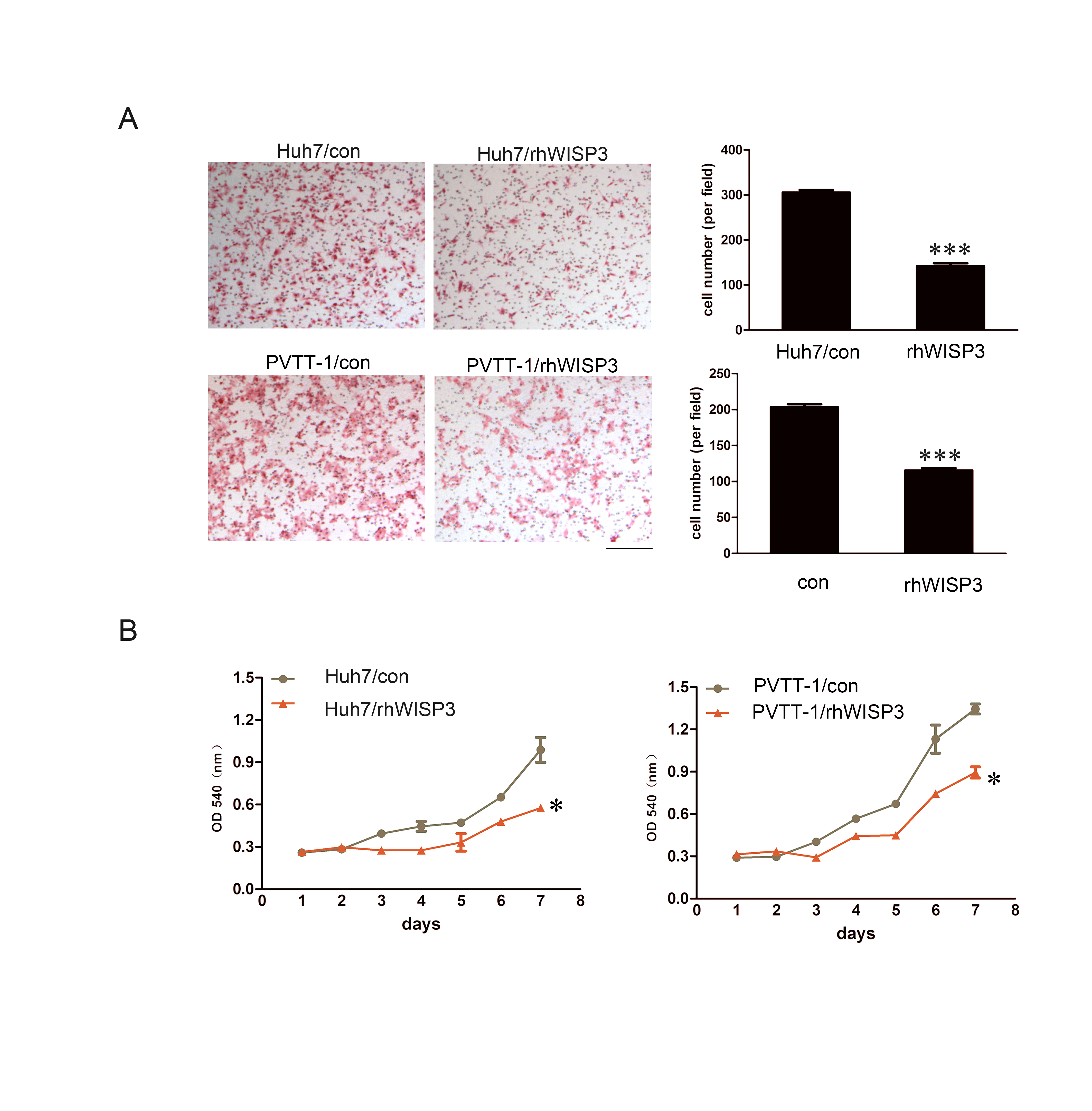

Supplement: Supplementary file 1 [file CPR-52-e12583-s001.tif]

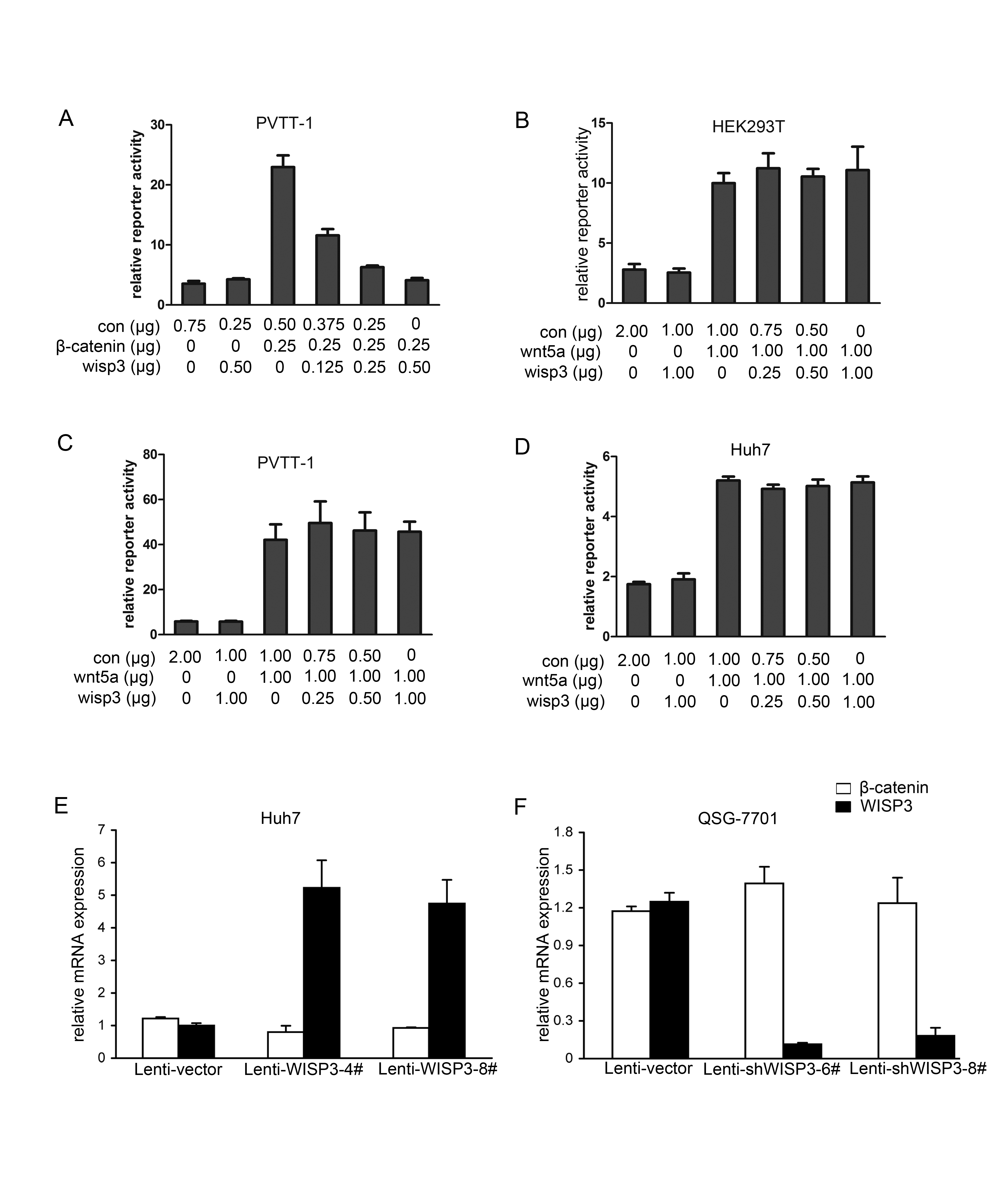

Supplement: Supplementary file 2 [file CPR-52-e12583-s002.tif]

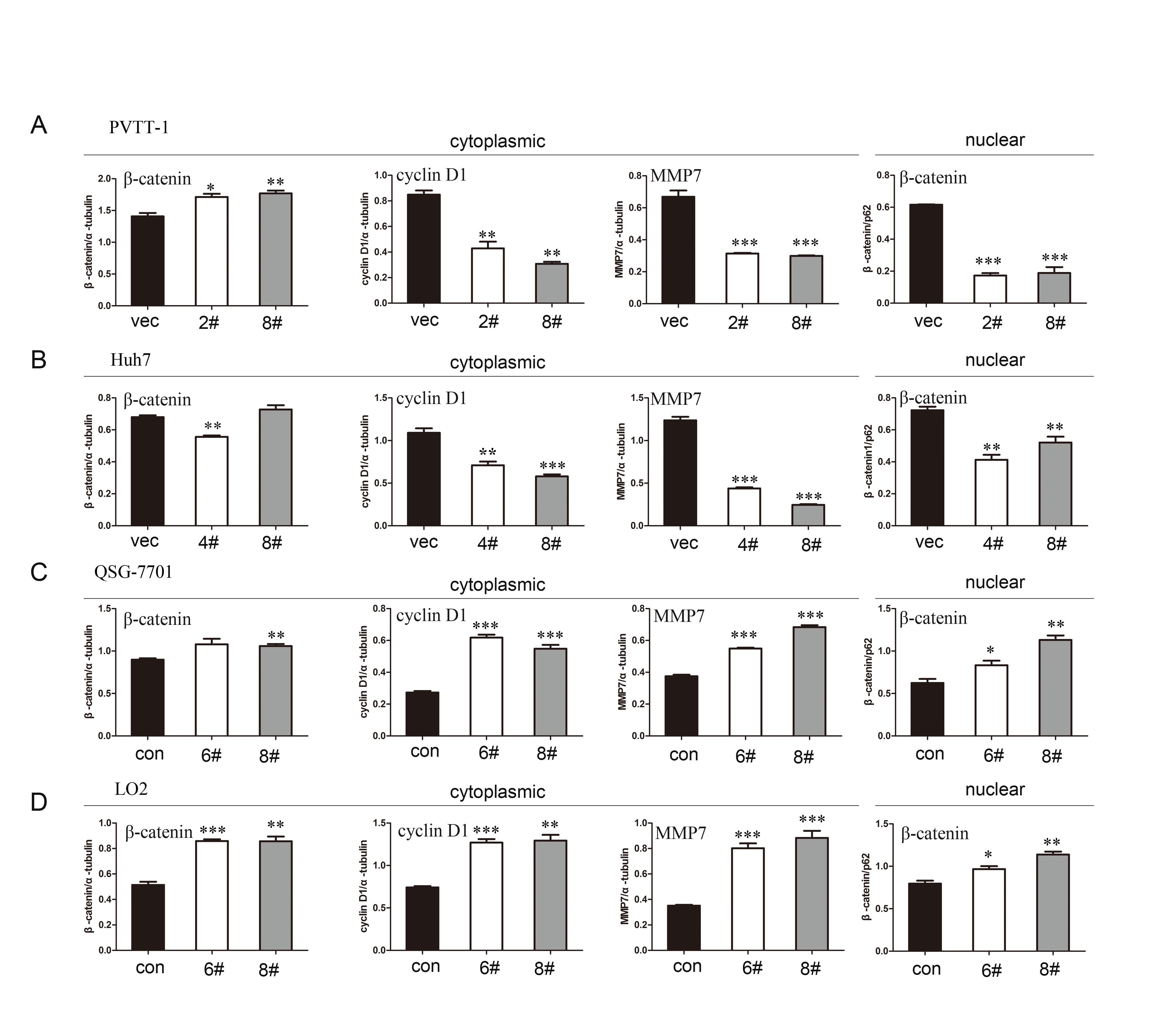

Supplement: Supplementary file 3 [file CPR-52-e12583-s003.tif]

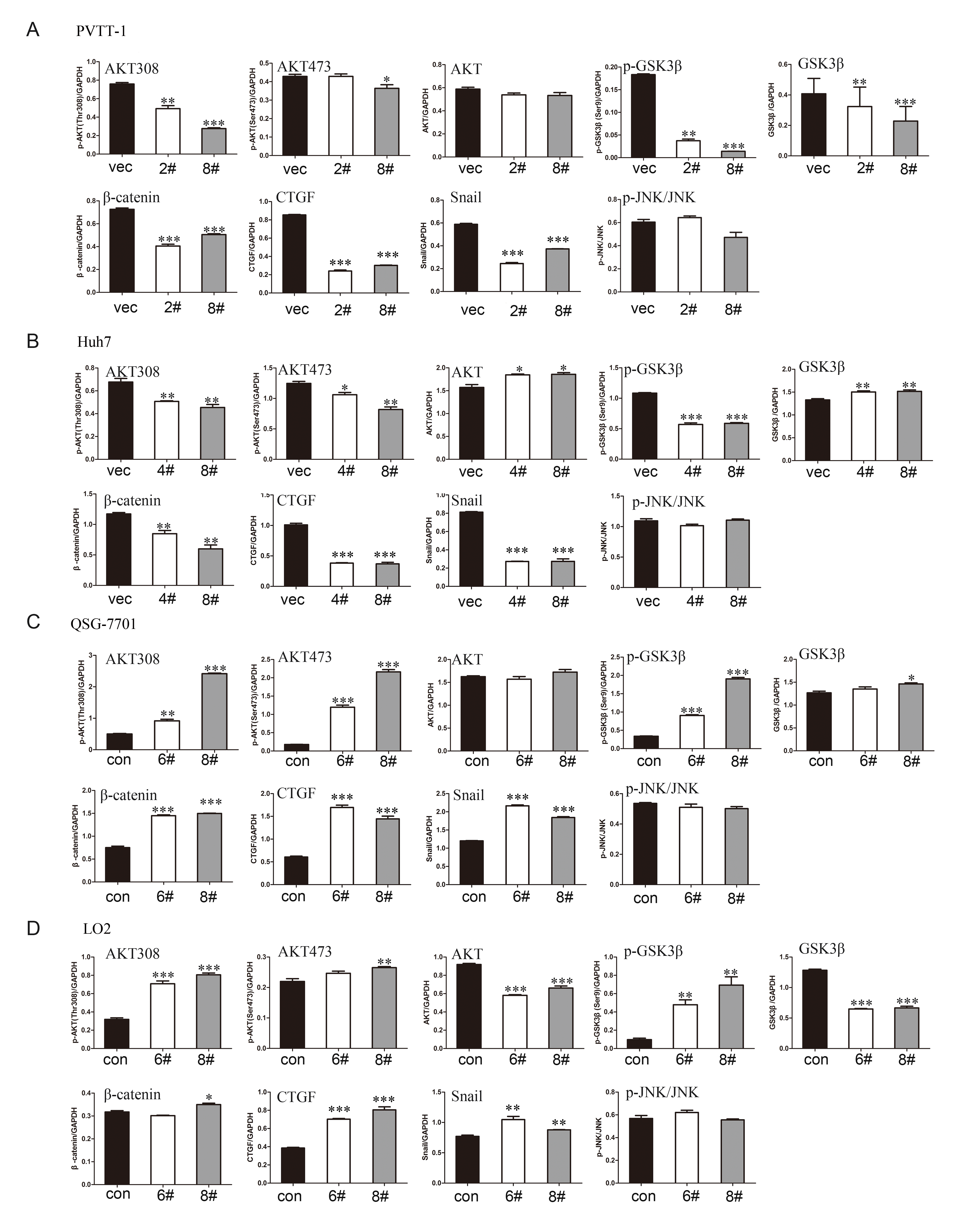

Supplement: Supplementary file 4 [file CPR-52-e12583-s004.tif]

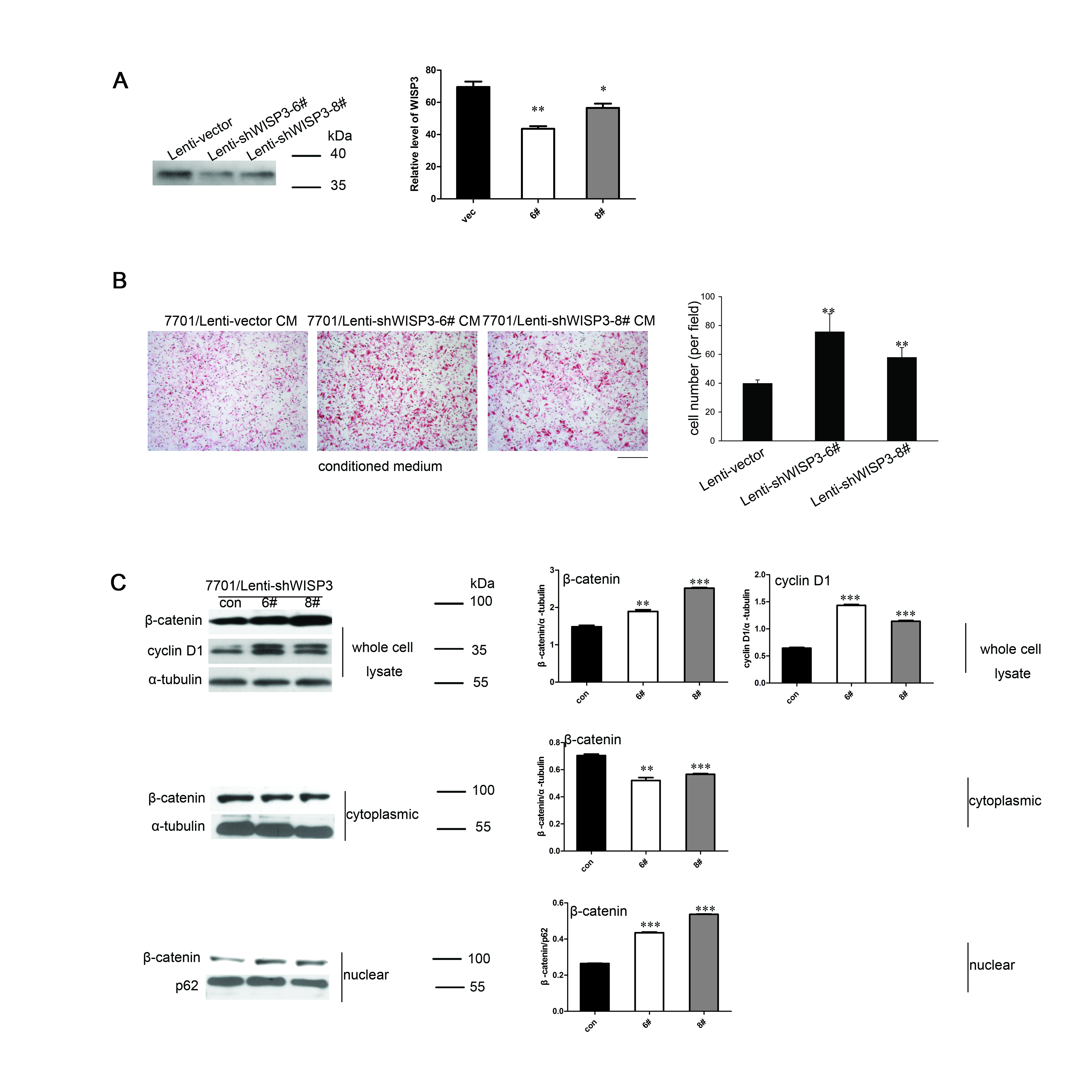

Supplement: Supplementary file 5 [file CPR-52-e12583-s005.tif]

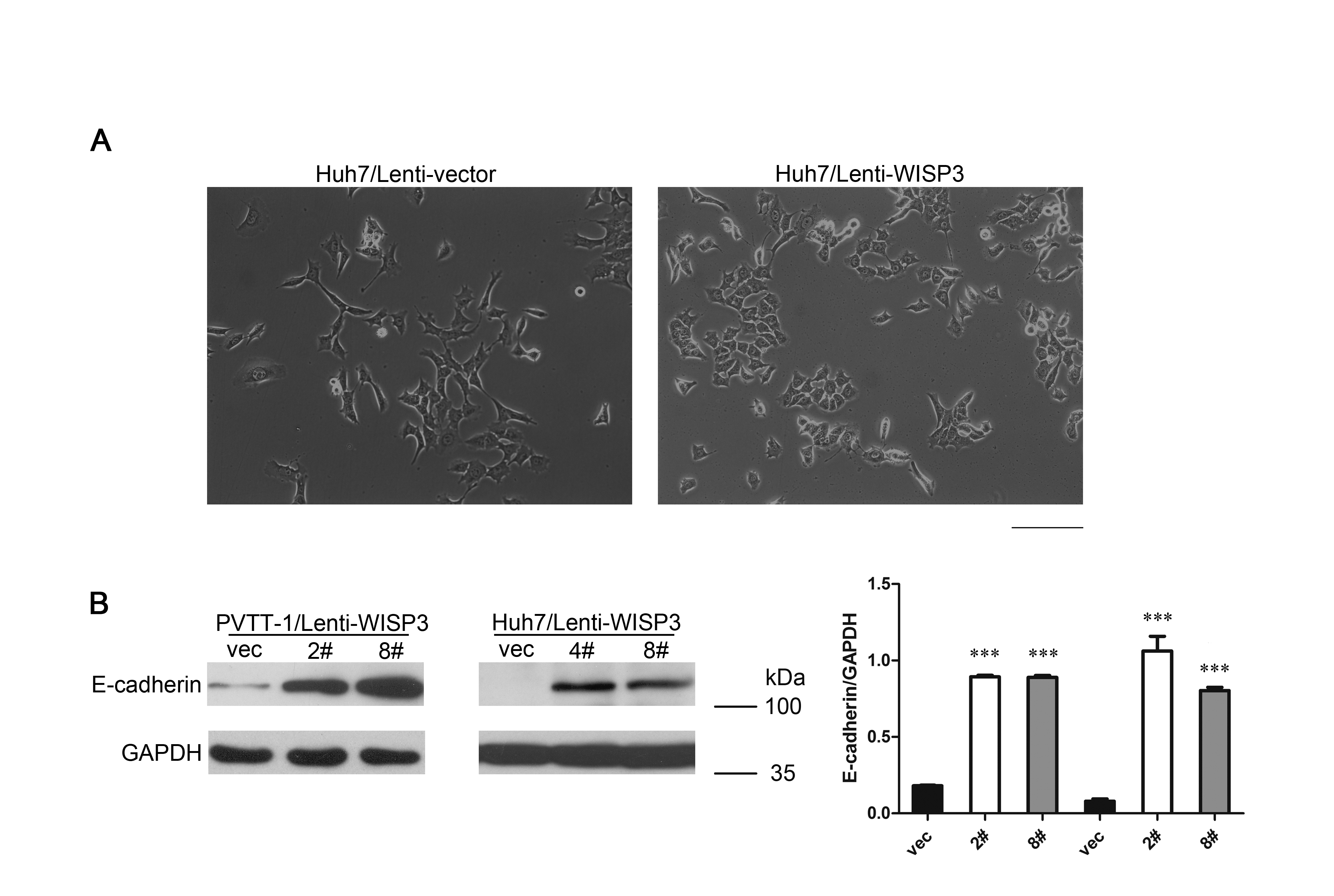

Supplement: Supplementary file 6 [file CPR-52-e12583-s006.tif]
