# Supplementary material for: Liver cancer: WISP3 suppresses hepatocellular carcinoma progression by negative regulation of β‐catenin/TCF/LEF signalling
Source: Cell Prolif. 2019 Feb 22;52(3):e12583. doi: 10.1111/cpr.12583 (PMC6536422; doi:10.1111/cpr.12583)
Supplement: Supplementary file 7 [file CPR-52-e12583-s007.doc]

|  | No. | mRNA |  |
| --- | --- | --- | --- |
| Characteristic | of patients | (mean±SE)^a^ | *p* |
| **Gender** |  |  |  |
| Male | 63 | -1.66±0.41 | 0.216 |
| Famle | 8 | -3.17±0.93 |  |
| **Age (years)** |  |  |  |
| ≤51 | 47 | -1.94±0.49 | 0.7 |
| ＞51 | 24 | -1.62±0.63 |  |
| **Hepatitics Status** |  |  |  |
| No | 3 | -0.55±0.26 | 0.49 |
| Yes | 68 | -1.89±0.4 |  |
| **Liver Cirrhosis** |  |  |  |
| No | 5 | -2.11±0.77 | 0.84 |
| Yes | 66 | -1.8±0.41 |  |
| **AFP (ng/mL)** |  |  |  |
| ≤20 | 14 | -0.14±1.19 | 0.02 |
| ＞20 | 57 | -2.34±0.31 |  |
| **Tumor Size (cm)** |  |  |  |
| ≤5 | 12 | 0.12±1.26 | 0.02 |
| ＞5 | 59 | -2.22±0.37 |  |
| **Tumor Number** |  |  |  |
| Single | 45 | -1.98±0.43 | 0.617 |
| Multiple | 26 | -1.58±0.75 |  |
| **Tumor Capsulation** |  |  |  |
| Complete | 16 | -2.7±0.55 | 0.24 |
| Incomplete | 55 | -1.6±0.46 |  |
| **Vascular invasion** |  |  |  |
| No | 15 | -1.63±0.81 | 0.79 |
| Yes | 56 | -1.88±0.44 |  |
| **Lymphnode metastasis** |  |  |  |
| No | 64 | -1.82±0.42 | 0.91 |
| Yes | 7 | -1.97±0.54 |  |
| **Child-Pugh score** |  |  |  |
| A | 24 | -1.98±0.50 | 0.70 |
| B | 49 | -1.69±0.59 |  |
| **TNM stage** |  |  |  |
| Ⅰ/Ⅱ | 12 | -1.75±0.95 | 0.93 |
| Ⅲ/Ⅳ | 59 | -1.84±0.42 |  |

Table S1 The correlation between the mRNA level of WISP3 and the clinical features of the HCC Patients.

Note: a, the value is the relative levels of WISP3 in cancer tissues versus matched normal tissues normalized by β-actin. TNM, tumor-node-metastasis.
